# A facile and efficient method for the synthesis of crystalline tetrahydro-β-carbolines via the Pictet-Spengler reaction in water

**DOI:** 10.1038/s41598-020-57911-0

**Published:** 2020-01-23

**Authors:** Hong-Ju Byeon, Kyung-Hwan Jung, Gi-Seong Moon, Sung-Kwon Moon, Hyang-Yeol Lee

**Affiliations:** 1 Department of Biotechnology, Korea National University of Transportation, 61 Daehak-ro, Jeungpyeong-gun, Chungbuk 27909 Republic of Korea; 2 Department of Food and Nutrition, Chung-Ang University, 4726 Seodong-Daero, Daedeok-Myeon, Anseong 456-756 Republic of Korea

**Keywords:** Biological techniques, Cancer therapy, Drug discovery and development

## Abstract

A facile and efficient synthesis of tetrahydro-β-carbolines (tryptolines) in one step from tryptamine and aldehydes, in an environmentally friendly water solvent, has been investigated. This convenient and clean synthesis of various tryptolines was facilitated by l-tartaric acid, a natural compound, to obtain the desired products as clear crystals. Among the four crystalline products, the most substituted tryptoline **2** showed the best inhibitory activity against EJ cells and the least cytotoxicity, with an LC_50_ value of 1.49 mg/mL, against brine shrimp larvae.

## Introduction

The tetrahydro-β-carboline (tryptoline) moiety in the products of the Pictet-Spengler reaction has been observed in various synthetic and natural compounds. Tryptolines have been known to have many pharmacological and biological activities such as antimicrobial, antitumor, antiviral, and anticonvulsant activities, which has led to increased interest in the synthesis of these compounds^1–4^. Enantioselective tryptolines would be important for accessing useful chiral building blocks in complex alkaloid synthesis^5^. A number of enantioselective synthetic methods have been reported. Chirality can be introduced to the desired product by using asymmetric reduction methods using Noyori-type catalysis^6^. Better success has been achieved with Brönsted acid catalysts. Asymmetric catalysis of tryptoline products has been developed with N-acyliminium ions^7^ or N-sulfenyliminium ions^8^. More recently, chiral thiourea derivatives in combination with benzoic acid promote catalytic asymmetric Picted-Spengler reaction to provide unprotected tryptolines in high ee and yield^9^. However, most of the reported synthetic methods require organic solvents and expensive or hazardous catalysts^10–12^. Thus, the use of organic solvents in industrial organic reactions is limited because the presence of water in these solvents often results in low yields of the products and/or low reaction rates. Moreover, due to concerns over human health and the environment, environmentally friendly reactions have attracted greater attention from the viewpoint of green chemistry as compared to chemical processes that use organic solvents and toxic reagents. Thus, aqueous reactions are desirable since water is the safest and most inexpensive green solvent^13–16^. Aqueous reactions for the Pictet-Spengler cyclization are known, but they require harsh conditions or a large excess of strong acid catalysts. A variety of efficient catalytic systems, especially those based on Lewis or Brönsted acids, have been developed for these aqueous reactions, but most of the systems are expensive or hazardous^7,17–19^. Moreover, the products formed are racemic mixtures^20^.

In this study, we report a naturally abundant compound, l-tartaric acid, which facilitates the aqueous Pictet-Spengler reactions and affords the products as colorless crystals. l-tartaric acid facilitates the formation of the desired tryptolines **1–4** under mild conditions, in the absence of an organic solvent. This new method using water and l-tartaric acid is safe, inexpensive, and easy, and the tryptolines can be conveniently isolated by simple filtration as they are formed as crystals. The anticancer activity of tryptolines **1–4** against EJ cells was investigated, and their cytotoxicity was determined by the brine shrimp lethality assay^21^.

## Results

**Figure 1:**
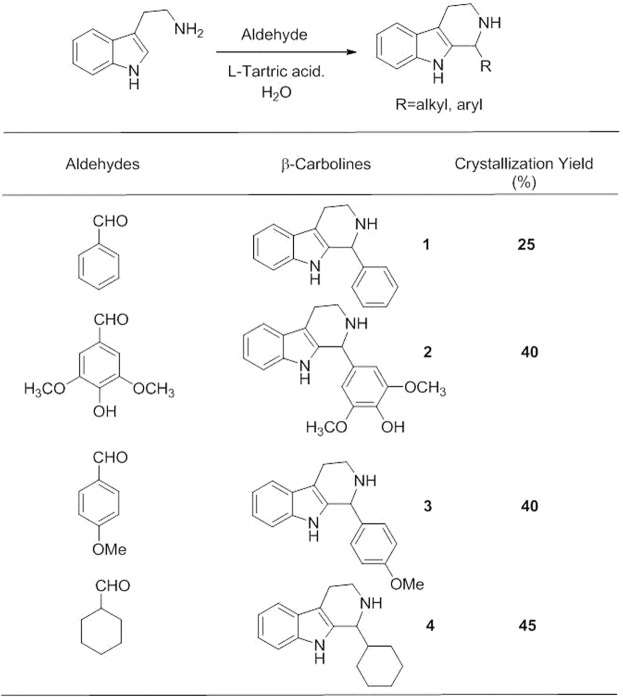
Scope of the aqueous Pictet-Spengler reaction using tryptamine and four different aldehydes facilitated by l-tartaric acid. **(1)** 1-phenyl-2,3,4,9-tetrahydro-1*H*-pyrido[3,4-*b*]indole, **(2)** 2,6-dimethoxy-4-(2,3,4,9-tetrahydro-1*H*-pyrido[3,4-*b*]indol-1-yl)phenol, (**3**) 1-(4-methoxyphenyl)-2,3,4,9-tetrahydro-1H-pyrido[3,4-*b*]-indole, and (**4**) 1-cyclohexyl-2,3,4,9-tetrahydro-1*H*-pyrido[3,4-*b*]indole.

**Figure 2:**
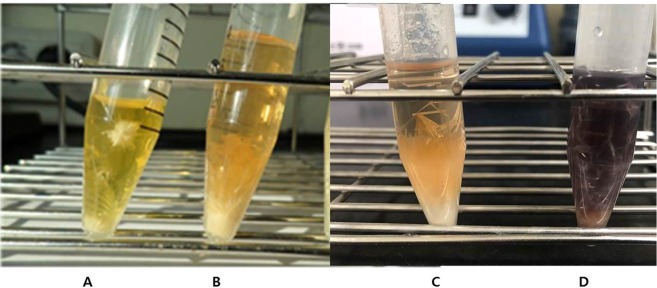
Crystals of tryptolines obtained from the aqueous Pictet-Spengler reaction of tryptamine with four different aldehydes facilitated by l-tartaric acid; (**A**) (**1**), (**B**) (**4**), (**C**) (**2**), (**D**) (**3**).

We examined the substrate scope of the Pictet-Spengler reaction with four different aldehydes, as shown in Fig. 1. l-Tartaric acid facilitated the formation of colorless-crystalline tryptolines, as shown in Fig. 2. The optimum amount of l-tartaric acid was determined by carrying out the experiment with 0.1–1 equiv. of l-tartaric acid. We found that 0.5 equiv. of l-tartaric acid was sufficient to give the highest yield, as the yield did not improve when using more than 0.5 equiv. of l-tartaric acid. The reaction was carried out in water, which is a cheap and safe solvent. The reaction mixture was set still until the crystals formed, and the products were filtered without any additional workup. Tryptolines **1–4** were obtained in crystalline form and in fair yields (25–45%), and required no chromatography. The most substituted β-carboline **4** showed the best inhibitory activity against EJ cells.

### 1-Phenyl-2,3,4,9-tetrahydro-1*H*-pyrido[3,4-*b*]indole (1)

Yield: 25% (crystalline). [α]^25^_D_ = −0.48 (c = 0.5, MeOH). Mp. 240 ~ 243 °C. ^**1**^**H NMR** (500 MHz, MeOD) *δ* (ppm): 7.59 (d, 1H, *J* = 8.0 Hz), 7.56-7.53 (m, 3H), 7.48-7.46 (m, 2H), 7.33 (d, 1H, *J* = 8.0 Hz), 7.19 (t, 1H, *J* = 8.0 Hz), 7.12 (t, 1H, *J* = 8.0 Hz), 5.96 (s, 1H), 3.70-3.65 (m, 1H), 3.62-3.57 (m, 1H), 3.35-3.26 (m, 1H), 3.22-3.18 (m, 1H). ^**13**^**C NMR** (100 MHz, MeOD) *δ* (ppm): 137.1, 135.3, 130.6, 130.2, 129.3, 129.1, 126.3, 122.4, 119.5, 118.7, 112.1, 108.0, 56.0, 18.6. Microscope-FTIR (KBr pellet, cm^−1^) 3213 (s), 3051(w), 2904 (s), 2804 (s), 2748 (s), 2692 (s), 2675 (s), 2611 (s), 2571 (s), 2480 (w). **LCMS** [M + H]^+^ *m/z* calcd. for C_17_H_17_N_2_ 249.1392; detected 249.1315.

### 2,6-Dimethoxy-4-(2,3,4,9-tetrahydro-1H-pyrido[3,4-b]indol-1-yl)phenol (2)

Yield: 40% (crystalline). [α]^25^_D_ = −2.76 (c = 0.5, MeOH). Mp. 206~210 °C.

^**1**^**H NMR** (500 MHz, MeOD) *δ* (ppm): 7.58 (d, 1H, *J* = 9.0 Hz), 7.34 (d, 1H, *J* = 8.0 Hz), 7.19 (t, 1H, *J* = 8.0 Hz), 7.12 (t, 1H, *J* = 8.0 Hz), 6.74 (s, 2H), 5.86 (s, 1H), 3.84 (s, 6H), 3.74-3.70 (m, 1H), 3.62-3.57 (m, 1H), 3.31-3.26 (m, 1H), 3.21-3.16 (m, 1H). ^**13**^**C NMR** (100 MHz, MeOD) *δ* (ppm):): 163.6, 139.3, 133.1, 129.7, 128.1, 128.0, 124.5, 121.5, 120.1, 116.6, 113.3, 109.7, 58.8, 56.8, 42.9, 20.4. Microscope-FTIR (KBr pellet, cm^−1^) 3433 (w), 3234 (w), 3059 (w), 2943 (w), 2902 (w), 2800 (s), 2646 (w), 1618 (s), 1523 (s), 1466 (s), 1427 (s), 1336 (s), 1238 (s), 1119 (s). **LCMS** [M + H]^+^ *m/z* calcd. for C_19_H_21_N_2_O_3_ 325.1552; detected 325.1460.

### 1-(4-Methoxyphenyl)-2,3,4,9-tetrahydro-1H-pyrido[3,4-b]–indole (3)

Yield: 40% (crystalline). [α]^25^_D_ = −2.04 (c = 0.5, MeOH). Mp. 242~244 °C.

^**1**^**H NMR** (500 MHz, MeOD) *δ* (ppm): 7.57 (d, 1H, *J* = 9.0 Hz), 7.33 (d, 1H, *J* = 8.0 Hz), 7.19 (t, 1H, *J* = 7.0 Hz), 7.12 (t, 1H, *J* = 7.0 Hz), 7.07 (d, 2H, *J* = 9.0 Hz), 5.89 (s, 1H), 3.86 (s, 3H), 3.67-3.63 (m, 1H), 3.59-3.54 (m, 1H), 3.11-3.07 (m, 1H), 3.30-3.24 (m, 1H), 3.20-3.15 (m, 1H). ^**13**^**C NMR** (100 MHz, MeOD) *δ* (ppm) 155.9, 137.1, 131.4, 130.3, 122.3, 120.2, 119.6, 119.2, 117.8, 115.2, 111.1, 107.6, 51.4, 39.8, 18.3. Microscope-FTIR (KBr pellet, cm^−1^) 3222 (s), 3014 (w), 2902 (s), 2790 (s), 2742 (s), 2567 (w), 1614 (s), 1516 (s), 1454 (s), 1430 9 s), 1307 (s), 1254 (s), 1180 (s), 741 (s). **LCMS** [M + H]^+^ *m/z* calcd. for C_19_H_19_N_2_O 279.1497; detected 279.1364.

### 1-Cyclohexyl-2,3,4,9-tetrahydro-1H-pyrido[3,4-b]indole (4)

Yield: 45% (crystalline). [α]^25^_D_ = −3.08 (c = 0.5, MeOH). Mp. 242~246 °C. ^**1**^**H NMR** (500 MHz, MeOD) *δ* (ppm): 7.51 (dd, 1H, *J* = 8.0 Hz, *J* = 2.5 Hz), 7.40 (d, 1H, *J* = 8.0 Hz), 7.18 (t, 1H, *J* = 7.0 Hz), 7.08 (td, 1H, *J* = 7.0 Hz, 0.5 Hz), 4.65 (d, 1H, *J* = 4.5 Hz), 3.79-3.74 (m, 1H), 3.47-3.42 (m, 1H), 3.18-3.11 (m, 1H), 3.11-3.07 (m, 1H), 2.35-2.29 (m, 1H), 1.95 (t, 2H, *J* = 10.5 Hz), 1.82 (t, 2H, *J* = 16.0 Hz), 1.53 (t, 1H, *J* = 10.0 Hz), 1.48-1.38 (m, 1H), 1.32-1.20 (m, 2H). ^**13**^**C NMR** (100 MHz, MeOD) *δ* (ppm): 137.3, 130.0, 126.8, 122.5, 119.8, 118.6, 112.3, 107.7, 58.2, 40.8, 30.2, 27.0, 26.7, 26.6, 18.9. Microscope-FTIR (KBr pellet, cm^−1^) 3219 (s), 3053 (w), 2933 (s), 2856 (m), 2763 (m), 1620 (s), 1454 (s), 1429 (s), 1307 (s), 1221 (s). **LCMS** [M + H]^+^ *m/z* calcd. for C_17_H_23_N_2_ 255.1861; detected 255.1789.

**Figure 3:**
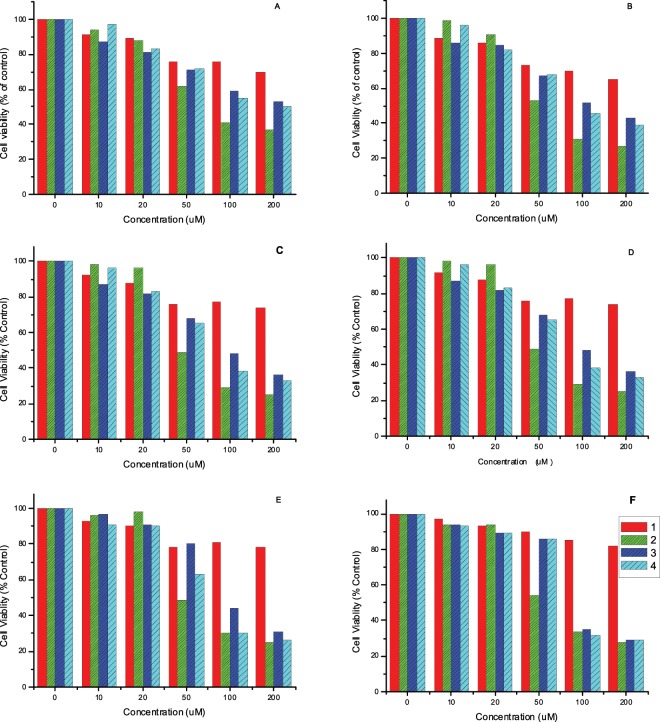
MTT assay to evaluate EJ cell inhibition potency of tryptolines **1**–**4** (red: **1**, green: **2**, blue: **3**, and sky blue: **4**) treated for (**A**) 12 h, (**B**) 24 h, (**C**) 36 h, (**D**) 48 h, (**E**) 72 h, and (**F**) 96 h.

The greater the substitution on the benzyl ring of the product, the better its potency to inhibit the growth of the EJ cells. Therefore, the most substituted tryptoline **2** showed the highest inhibitory activity against the EJ cells, as shown in Fig. 3. We speculated that tryptolines may need a bulky substituent for better binding affinity to the hydrophobic pocket of the target protein. This assumption is consistent with the fact that the clinically used anticancer drugs vinblastine and vincristine are dimers of tryptolines and are thus bulkier than monomeric tryptolines.

**Figure 4:**
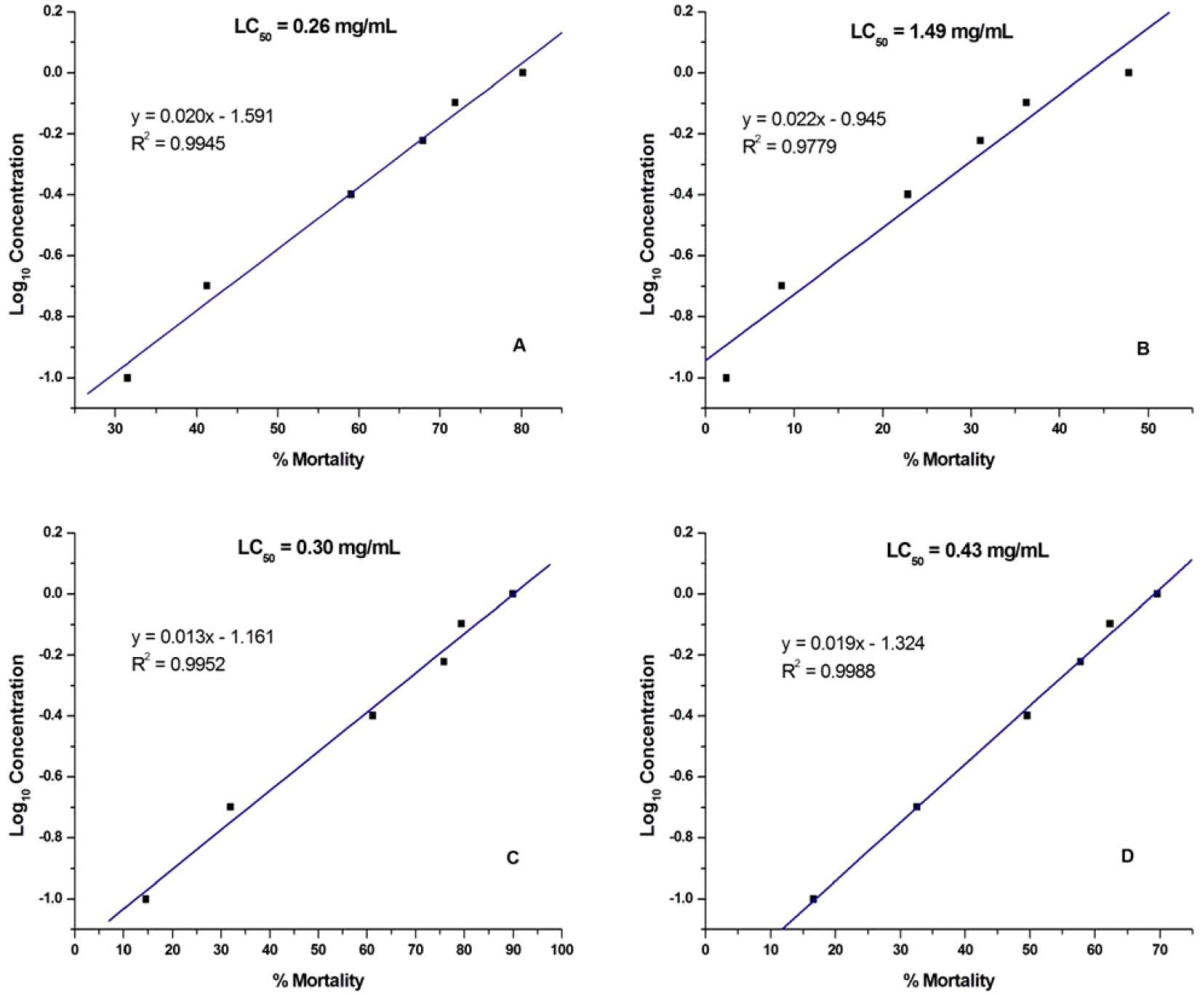
Cytotoxic effects of tryptolines **1(A)–4 (D)** on brine shrimp larvae for an incubation period of 24 h, determined using the brine shrimp lethality assay.

The cytotoxicity results for tryptolines **1**–**4** against brine shrimp larvae are shown in Fig. 4. Tryptolines **1**, **3**, and **4** showed weak cytotoxicity, with LC_50_ values of 0.26 mg/mL, 0.30 mg/mL, and 0.43 mg/mL, respectively. However, **2**, which showed the highest inhibitory activity against the EJ cells, exhibited the least cytotoxicity with an LC_50_ value of 1.49 mg/mL. Tryptoline **2** showed no significant cytotoxicity (LC_50_ > 1.0 mg/mL) against brine shrimp larvae, as opposed to the positive control (K_2_Cr_2_O_7_), which showed significant cytotoxicity with an LC_100_ value of 0.1 mg/mL, over incubation period of 24 h. Interestingly, **2** showed the highest potency against the EJ cells but the least cytotoxicity as compared to **1**, **3**, and **4**.

## Discussion

In conclusion, a facile method for the synthesis of biologically active tryptolines using l-tartaric acid, a simple and natural compound, is developed. l-tartaric acid facilitated aqueous Pictet-Spengler reactions with four different aldehydes. Moreover, the colorless crystalline tryptolines formed could be directly filtered to avoid further isolation steps such as extractions, column chromatography or HPLC. All of the major crystalline products showed (S)-conformer as a dominant species, based on the optical rotations we measured, although these reactions were not highly enantioselective. With respect to the anti-cancer activity of the crystalline products **1**~**4**, compound **2** showed the best cytotoxicity against EJ cell line. We believe that this is one of the safest and easiest methods to prepare the key intermediates in the first step of the synthesis of biologically active vinca alkaloids, especially at the industrial-scale.

## Methods

### General procedure for the synthesis of tryptolines

l-Tartaric acid (0.5 equiv., 0.5 mmol) was added to a mixture of tryptamine hydrochloride (1.0 equiv., 1.0 mmol) and aldehydes (1.0 equiv., 1.0 mmol) in a 15 mL falcon tube. The reaction volume was adjusted to 4.0 mL with water, and the tube was sealed. The tube was placed in a hot water bath at about 60 °C and set still for 1–2 days until crystals were formed. The crystals were then filtered, followed by rinsing with cold water and ether.

### High-resolution UPLC/QTOF/MS analysis

Ultra-performance LC/MS analysis was performed on an ultra-high-resolution Q-TOF LC-MS/MS system (Micro QTOF III, Bruker Co.) using a C18 column, with a particle size of 1.7 mm, dimensions of 2.1 × 100 mm, and flow rate of 0.6 mL min^−1^, and an electrospray ionization (ESI) source.

### MTT assay

EJ cells (3 × 10^5^ cells/well) were seeded in 6-well plates, followed by treatment with the indicated concentrations of the compounds. After 24 h, the used medium was removed, and a fresh medium containing 10 μL of MTT solution (5 mg/mL) was added to the wells, which were then allowed to stand for 1 h. Subsequently, the medium was removed, and the cells were treated with 0.1 mL of dimethyl sulfoxide (DMSO). The absorbance was measured at a wavelength of 540 nm using a microplate reader. The cell viability was expressed as the percentage of the absorbance value determined for the control cultures.

### Brine shrimp lethality bioassay

The brine shrimp lethality bioassay was carried out to determine the cytotoxicity of the tryptolines (**1–4**). The concentration that killed 50% of the shrimps (LC_50_) was determined, followed by the known method [17]. Two sets of these experiments were carried out in triplicate.

## Supplementary information

Supplementary Information.

## Data Availability

The datasets used and analyzed during the current study are available from the corresponding author on reasonable request. Complete experimental procedures and characterization data for products are in Supporting Information.
