# Supplementary material for: A facile and efficient method for the synthesis of crystalline tetrahydro-β-carbolines via the Pictet-Spengler reaction in water
Source: Sci Rep. 2020 Jan 23;10:1057. doi: 10.1038/s41598-020-57911-0 (PMC6978303; doi:10.1038/s41598-020-57911-0)
Supplement: Supplementary file 1 — Supplementary Information. [file 41598_2020_57911_MOESM1_ESM.docx]

**A facile and efficient method for the synthesis of crystalline tetrahydro-β-carbolines via the Pictet-Spengler reaction in water**

Hong-Joo Byun^a^ ‧ Kyung-Hwan Jung^a^ ‧ Gi-Seong Moon^a^ ‧Sung-Kwon Moon^b^ and Hyang-Yeol Lee^a^*

^a^*Department of Biotechnology, Korea National University of Transportation, 61 Daehak-ro, Jeungpyeong-gun, Chungbuk 27909, Republic of Korea

(<Tel:82-43-820-5252>, Fax:82-43-820-5252, E-mail: [hyl@ut.ac.kr](mailto:hyl@ut.ac.kr))

^b^Department of Food and Nutrition, Chung-Ang University, 4726 Seodong-Daero, Daedeok-Myeon, Anseong 456-756, Republic of Korea

(Tel: 82-31-670-3284, Fax: 82-31-675-4853, Email: sumoon66@cau.ac.kr)

S. K. Moon (🖂 )‧H. Y. Lee (🖂 )

S. K. Moon and H. Y. Lee contributed equally

**Supplemental Information**

- 1. **Brine shrimp lethality bioassay**
  2. *Hatching shrimp*

Brine shrimp (*Artemia salina*) eggs were hatched in seawater prepared by dissolving 38 g of natural sea salt in 1 L of water. After a 24 h incubation period at room temperature, the shrimp larvae were attracted to one side of the sample bottle using a light source, and 10–15 of them per 100 μL were collected with a micropipette.

- 1. *Brine shrimp lethality bioassay*

The cytotoxicity of the tryptolines (**1–4**) was monitored by the brine shrimp lethality bioassay. The tryptolines were dissolved in DMSO and diluted with seawater to a concentration of 0.1 to 1.0 mg/mL. A negative control was prepared using sea water and DMSO. A positive control was prepared by dissolving potassium dichromate in seawater to a concentration range of 0.1–1.0 mg/mL. Then, 0.1 mL of the shrimp suspension containing 10–15 shrimp larvae was added to 0.9 mL of each sample solution in a 48-well plate and incubated for 24 h. The plate was then examined, and the number of dead shrimps were counted in each well. A graph of the mean percentage mortality versus the logarithm of the concentrations was plotted, and the concentration that killed 50% of the shrimps (LC_50_) was determined (Finney 1971). Two sets of these experiments were carried out in triplicate.

**2. NMR analysis**

|  |
| --- |
| A |
|  |
| B |
| Figure. S1. NMR spectra of compound 1.  A: ^1^H-NMR, B: ^13^C-NMR. * NMR solvent: MeOD |

|  |
| --- |
| A |
|  |
| B |
| Figure. S2. NMR spectra of compound **2**.  A: ^1^H-NMR, B: ^13^C-NMR. *NMR solvent: MeOD |
| 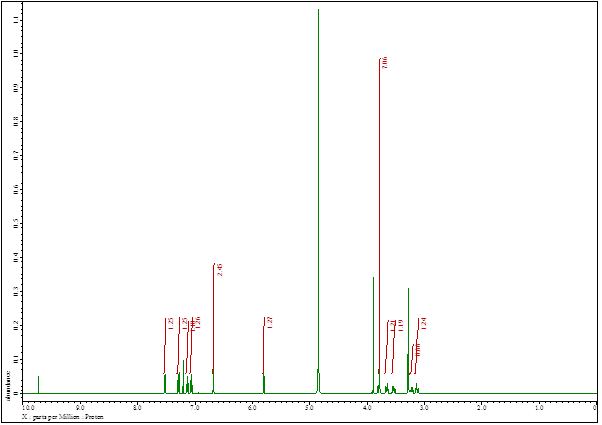 |
| A |
|  |
| B |
| Figure. S3. NMR spectra of compound **3**.  A: ^1^H-NMR, B: ^13^C-NMR. *NMR solvent: MeOD |

|  |
| --- |
| A |
|  |
| B |
| Figure. S4. NMR spectra of compound **4**.  A: ^1^H-NMR, B: ^13^C-NMR. *NMR solvent: MeOD |

**3. LC/MS Analysis**

| A |
| --- |
| B |
| C |
| D   |
| Figure. S5. UPLC/MS/QTOF spectra of β-carboline compounds. A: **1**, B: **2**, C: **3**, D: **4**. |

4. MTT assay


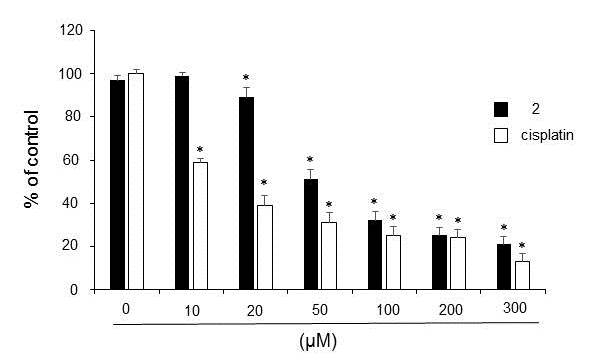


Figure. S6. MTT assay with cisplatin and compound **2** against EJ cell.
